# Supplementary material for: Exact low-temperature series expansion for the partition function of the zero-field Ising model on the infinite square lattice
Source: Sci Rep. 2016 Oct 10;6:33523. doi: 10.1038/srep33523 (PMC5056370; doi:10.1038/srep33523)
Supplement: Supplementary Information [file srep33523-s1.pdf]

Exact low-temperature series expansion for the partition function of the zero-field Ising model on the infinite square lattice Supplementary Information

Grzegorz Siudem,[1, a)] Agata Fronczak,[1, b)] and Piotr Fronczak[1, c)]
Faculty of Physics, Warsaw University of Technology, Koszykowa 75, PL-00-662 Warsaw, Poland

(Dated: 5 October 2016)

I. LOW TEMPERATURE SERIES EXPANSION OF f

By substituting

$$x = e^{-2\beta J}, \tag{1}$$

and

$$p = p(\theta_1, \theta_2) = \cos\theta_1 + \cos\theta_2, \tag{2}$$

into Eq. (6) in the main paper, the bulk free energy per site in the square lattice Ising model can be written as:

$$-\beta f(x) = \ln 2 + \frac{1}{8\pi^2}\int_0^{2\pi} d\theta_1 \int_0^{2\pi} d\theta_2 \ln\left[\left(\frac{x+x^{-1}}{2}\right)^2 - \frac{-x+x^{-1}}{2}p\right] \tag{3}$$

$$= \ln 2 + \frac{1}{8\pi^2}\int_0^{2\pi} d\theta_1 \int_0^{2\pi} d\theta_2 \ln\left[\frac{x^{-2}}{4}\left(x^4+2px^3+2x^2-2px+1\right)\right] \tag{4}$$

$$= \ln x^{-1} + \frac{1}{8\pi^2}\int_0^{2\pi} d\theta_1 \int_0^{2\pi} d\theta_2 \ln\left(1-2px+2x^2+2px^3+x^4\right). \tag{5}$$

Next, the integrand function in Eq. (5) can be decomposed into a Taylor series as:

$$\ln\left(1-2px+2x^2+2px^3+x^4\right) = \sum_{n=1}^{\infty} L_n(-2p, 2\cdot 2!, 2p\cdot 3!, 4!)\frac{x^n}{n!} \tag{6}$$

$$= \sum_{n=1}^{\infty}\frac{x^n}{n!}\sum_{k=1}^{n}(-1)^{k-1}(k-1)!B_{n,k}(-2p, 2\cdot 2!, 2p\cdot 3!, 4!), \tag{7}$$

where the so-called *logarithmic polynomials* have been used, which are defined as (see Eq. (5a), p. 140 in[1]):

$$\ln\left(\sum_{n=0}^{\infty} g_n \frac{x^n}{n!}\right) = \sum_{n=1}^{\infty} L_n\left(\{g_i\}\right)\frac{x^n}{n!} \tag{8}$$

$$= \sum_{n=1}^{\infty}\frac{x^n}{n!}\sum_{k=1}^{n}(-1)^{k-1}(k-1)!B_{n,k}\left(\{g_i\}\right), \tag{9}$$

where $B_{n,k}(\{g_i\})$ represent partial Bell polynomials, see Eq. (4) in the main paper.

Now, substituting Eq. (7) to (5) one gets the general expression for the low temperature series expansion of the

a) Electronic mail: siudem@if.pw.edu.pl
b) Electronic mail: agatka@if.pw.edu.pl
c) Electronic mail: fronczak@if.pw.edu.pl

bulk free energy per site (cf. Eq. (6) in the main paper):

$$-\beta f(x) = -\ln x + \sum_{n=1}^{\infty} a_n \frac{x^n}{n!}, \tag{10}$$

where the expansion coefficients are given by:

$$a_n = \frac{1}{8\pi^2} \sum_{k=1}^{n} (-1)^{k-1}(k-1)! \times \tag{11}$$
$$\times \int_0^{2\pi} d\theta_1 \int_0^{2\pi} d\theta_2 B_{n,k}(-2p, 2\cdot 2!, 2p\cdot 3!, 4!).$$

Eq. (11) can be further simplified by using the explicit formula for partial Bell polynomials, Eq. (4) in the main paper, according to which the polynomial $B_{n,k}$ in Eq. (11) can be written as:

$$B_{n,k}(-2p, 2\cdot 2!, 2p\cdot 3!, 4!)$$
$$= n! \sum_{d_1,d_2,d_3,d_4} \frac{(-2p)^{d_1}(2\cdot 2)^{d_2}(2p\cdot 3!)^{d_3}(4!)^{d_4}}{d_1!d_2!d_3!d_4!(1!)^{d_1}(2!)^{d_2}(3!)^{d_3}(4!)^{d_4}} \tag{12}$$
$$= n! \sum_{d_1,d_2,d_3,d_4} \frac{(-1)^{d_1} 2^{d_1+d_2+d_3}}{d_1!d_2!d_3!d_4!} p^{d_1+d_3}, \tag{13}$$

where the summation takes place over all integers $d_1, d_2, d_3, d_4 \geq 0$, such that

$$d_1 + d_2 + d_3 + d_4 = k, \tag{14}$$

and

$$d_1 + 2d_2 + 3d_3 + 4d_4 = n. \tag{15}$$

Now, after using Eqs. (13) and (14) in Eq. (11) one gets the following expression for a_n:

$$a_n = -\frac{n!}{8\pi^2} \sum_{d_1,d_2,d_3,d_4} \frac{(-1)^{d_2+d_3+d_4} 2^{d_1+d_2+d_3}}{(d_1+d_2+d_3+d_4)} \binom{d_1+d_2+d_3+d_4}{d_1,\, d_2,\, d_3,\, d_4} \int_0^{2\pi} d\theta_1 \int_0^{2\pi} d\theta_2 \, p^{d_1+d_3}, \tag{16}$$

where the explicit summation over k was omitted due to the fact that it is already included in the summation over the variables d_1, d_3, d_3, d_4 which now must only satisfy Eq. (15).

The last step towards the final expression for a_n is to show that the double integral in Eq. (16) simplifies to:

$$\int_0^{2\pi} d\theta_1 \int_0^{2\pi} d\theta_2 \, p^l = \begin{cases} 0 & \text{for odd } l, \\ 4\pi^2 \, 2^{-l} \binom{l}{l/2}^2 & \text{for even } l, \end{cases} \tag{17}$$

where p is given by Eq. (2). (For reasons of clarity, the detailed calculations leading to Eq. (17) are not discussed here, but will be discussed in Sect. II of this document.) Finally, by inserting Eq. (17) into (16) one gets Eqs. (7) and (8) which are in use in the primary article. For odd values of n:

$$a_n = 0, \tag{18}$$

and for even values of n:

$$a_n = \frac{n!}{2} \sum_{d_1,d_2,d_3,d_4} \binom{d_1+d_2+d_3+d_4}{d_1,\, d_2,\, d_3,\, d_4} \times \tag{19}$$
$$\times \frac{(-1)^{d_2+d_3+d_4-1} 2^{d_2}}{d_1+d_2+d_3+d_4} \binom{d_1+d_3}{\frac{d_1+d_3}{2}}^2,$$

where the summation takes place over all quadruple numbers $d_1, d_2, d_3, d_4 \geq 0$, which satisfy conditions $d_1 + 2d_2 + 3d_3 + 4d_4 = n$ and $d_1 + d_3$ is even.

II. DETAILED CALCULATIONS LEADING TO EQ. (17)

The double integral in Eq. (16) can be transformed as follows:

$$\int_0^{2\pi} d\theta_1 \int_0^{2\pi} d\theta_2 \, p^l \tag{20}$$
$$= 2^l \int_0^{2\pi} d\theta_1 \int_0^{2\pi} d\theta_2 \cos^l\left(\frac{\theta_1+\theta_2}{2}\right) \cos^l\left(\frac{\theta_1-\theta_2}{2}\right) \tag{21}$$
$$= 2^l \int_0^{2\pi} du \, \cos^l u \int_0^{2\pi} dv \, \cos^l v \tag{22}$$
$$= 2^l \left(\int_0^{2\pi} d\theta \, \cos^l \theta\right)^2 = 2^l \phi_l^2, \tag{23}$$

where the integral ϕ_l satisfies the below expression

$$\phi_l = \int_0^{2\pi} d\theta \, \cos^l \theta \tag{24}$$
$$= \cos^{l-1}\theta \sin\theta \Big|_0^{2\pi} + (l-1) \int_0^{2\pi} d\theta \, \cos^{l-2}\theta \, \sin^2\theta \tag{25}$$
$$= (l-1) \int_0^{2\pi} d\theta \, \cos^{l-2}\theta \left(1 - \cos^2\theta\right) \tag{26}$$
$$= (l-1)\phi_{l-2} - (l-1)\phi_l. \tag{27}$$

which leads to the following recursive equation:

$$\phi_l = \frac{l-1}{l}\,\phi_{l-2}, \qquad \text{for } l = 1, 2, 3\ldots, \tag{28}$$

with

$$\phi_0 = \int_0^{2\pi} d\theta = 2\pi \quad \text{and} \quad \phi_1 = \int_0^{2\pi} d\theta\,\cos\theta = 0. \tag{29}$$

Now, since the only solution of Eq. (28) is

$$\phi_l = 0 \quad \text{for odd} \quad l = 1, 3, 5, \ldots, \tag{30}$$

and

$$\phi_l = 2\pi\frac{(l-1)!!}{l!!} \quad \text{for even} \quad l = 0, 2, 4, \ldots, \tag{31}$$

Eq. (23) can be further simplified to:

$$\int_0^{2\pi} d\theta_1 \int_0^{2\pi} d\theta_2\; p^l = 2^l (2\pi)^2 \left(\frac{(l-1)!!}{l!!}\right)^2 \tag{32}$$

$$= 4\pi^2\, 2^l \left(\frac{l!}{l!!^2}\right)^2 \tag{33}$$

$$= 4\pi^2\, 2^l \left(\frac{l!}{2^l (l/2)!^2}\right)^2 \tag{34}$$

$$= 4\pi^2\, 2^{-l} \binom{l}{(l/2)}^2, \tag{35}$$

where the assumption that l is even has been used. Eq. (35) exactly corresponds to Eq. (17).

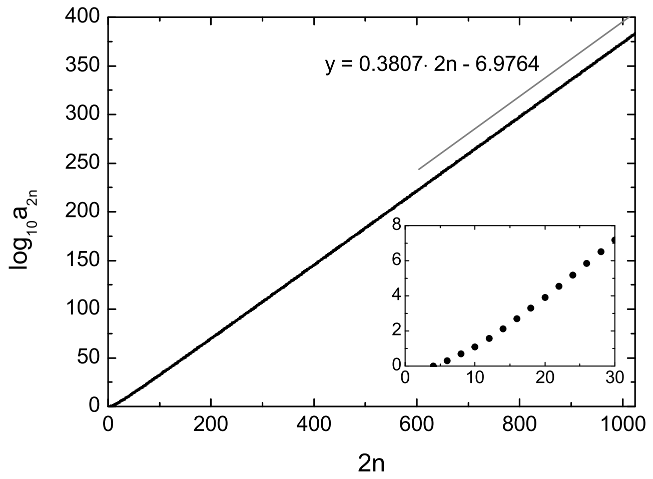

FIG. 1. Asymptotic behaviour of the sequence $a_{2n}/(2n)!$ vs $2n$.

III. ASYMPTOTIC BEHAVIOUR OF THE COEFFICIENTS $a_{2n}/(2n)!$

As we argued in the main paper the coefficients in the low temperature series expansion of $-\beta f(x)$ (see Eqs. (10), (18), and (19)) have the asymptotic behaviour which is given by Eq. (13) in the main paper:

$$\lim_{n\to\infty} \frac{a_{2n}}{(2n)!} = C\alpha^{2n}, \tag{36}$$

where C is a positive constant, and (cf. Eq. (20), in the main paper)

$$\alpha \simeq \frac{1}{x_c} = \exp\left[\frac{2J}{k_B T_c}\right] = \frac{1}{\sqrt{2}-1}. \tag{37}$$

The log-linear plot of the coefficients $a_{2n}/(2n)!$ vs $2n$, which is shown in Fig. 1 illustrates this behaviour. The logarithm of α:

$$\log_{10}\alpha \simeq 0.3828, \tag{38}$$

corresponds to the slope of the line, $a = 0.3807$,which is fitted to the results.

IV. EXACT ENERGY DISTRIBUTION $P(N, x)$ FOR THE SQUARE LATTICE ISING MODEL

Eq. (21) in the main paper, which is exact in the limit of infinite lattice size, i.e. for $V \to \infty$, provides an excellent testbed for comparison of the exact infinite-volume results and the results of finite-size Monte Carlo methods (see e.g.[2–4]).

In Fig. 2, the exact energy distribution $P(V, x)$, Eq. (21) in the main paper, is shown for three lattices of size: $V = 256,\ 512,\ 1024$, and two different temperatures: $x = e^{-2\beta J} = 0.36$ and 0.41 (let us note that $x_c \simeq 0.414$).

V. DETAILED CALCULATIONS LEADING TO EQ. (21) IN THE MAIN PAPER

By using Eq. (20) in the main paper and substituting r for $\frac{x}{x_c}$, the numerator in Eq. (19) in the main paper can be written as follows:

$$\frac{1}{N!}Y_N(\{a_n x^n\}) \simeq \frac{1}{N!}Y_N(\{Cr^n n!\}) \tag{39}$$

$$= \frac{1}{N!}\sum_{k=1}^{N} B_{N,k}(\{Cr^n n!\}) \tag{40}$$

$$= \frac{1}{N!}\sum_{k=1}^{N} C^k r^N B_{N,k}(\{n!\}), \tag{41}$$

where the expression (18) in the main paper has been used. Then, since the partial Bell polynomials with the coefficients: $1!, 2!, 3!\ldots$ are equal to Lah numbers,

$$B_{N,k}(1!, 2!, 3!, \ldots) = \frac{N!}{k!}\binom{N-1}{k-1}, \tag{42}$$

Eq. (41) can be further simplified:

$$\frac{1}{N!}Y_N(\{a_n x^n\}) \simeq r^N \sum_{k=1}^{N} \binom{N-1}{k-1}\frac{C^k}{k!} \tag{43}$$

$$\overset{N\gg 1}{\simeq} r^N C \sum_{l=0}^{\infty} \binom{N-1}{l}\frac{C^l}{(l+1)!} \tag{44}$$

$$= r^N C \sum_{l=0}^{\infty} \left((-1)^l \frac{(N-1)!}{(N-1-l)!}\right)\left(\frac{1}{(l+1)!}\right)\frac{(-C)^l}{l!} \tag{45}$$

$$= r^N C \sum_{l=0}^{\infty} \frac{(1-N)_k}{(2)_k}\frac{(-C)^l}{l!} \tag{46}$$

$$= r^N C \;\; {}_1F_1(1-N;2;-C), \tag{47}$$

where ${}_1F_1(1-N;2;-C)$ is the so-called confluent hypergeometric function of the first kind [26], which is defined as:

$${}_1F_1(a;b;z) = 1 + \frac{a}{b}z + \frac{a(a+1)}{b(b+1)}\frac{z^2}{2!} + \dots \tag{48}$$

$$= \sum_{k=0}^{\infty} \frac{(a)_k}{(b)_k}\frac{z^k}{k!}, \tag{49}$$

where $(a)_k$ and $(b)_k$ are Pochhammer symbols.

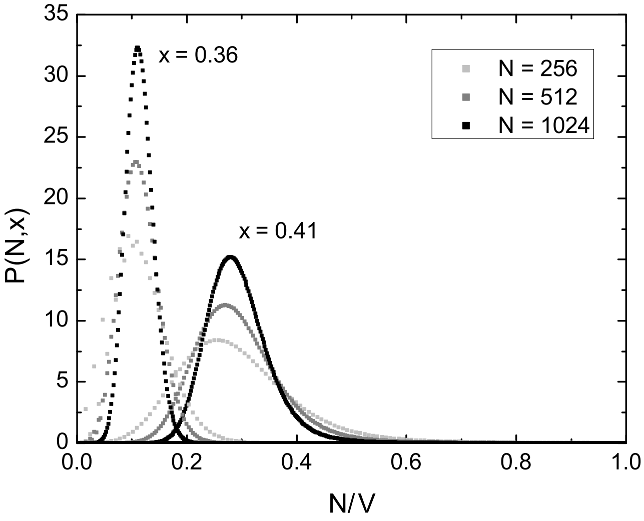

FIG. 2. Exact energy distribution $P(N,x)$ for the square lattice Ising model.

Finally, by substituting Eq. (47) to (19) in the main paper one gets the energy distribution as given by Eq. (21) in the main paper.

VI. MATHEMATICA ROUTINES

In this section we present our Mathematica scripts which allow to calculate coefficients of the low-temperature expansion of the free energy, {an}, and coefficients of the expansion of the partition function, {Yn}.

Since the built-in Mathematica `BellY[]` function for calculating Bell polynomials works very slow, we implement Bell polynomials using the following recurrence formula (Eq. (3k) in[1])

$$B_{n,k}(\{a_N\}) = \sum_{l=k-1}^{n-1} \binom{n}{l} a_{n-l} B_{l,k-l}(\{a_N\}).$$

Listing 1. The coefficients of the free energy

```
In[1]:=    (* list of sets {d1,d2,d3,d4}, where d1+2*d2+3*d3
     +4*d4=n and d1+d3 is even *)
       Belllist [n_] := Select[FrobeniusSolve[Range[4], n],
           EvenQ[#[[1]] + #[[3]]] &]
        (* function of m={d1,d2,d3,d4} under the sum in Eq
            .(20) *)
        ff[m_] := ((-1)^(m[[2]] + m[[3]] + m[[4]]) 2^m[[2]])/(
            m[[1]] + m[[2]] + m[[3]] + m[[4]])
            Multinomial[m[[1]], m[[2]], m[[3]], m[[4]]]
                Binomial[(m[[1]] + m[[3]]), (m[[1]] + m
                    [[3]])/2]^2
        (* final function for coefficients of free energy*)
        a[n_] := - (n!/2) If[OddQ[n], 0, Plus @@ (ff /@
            Belllist [n])];
        (*number of calculated coefficients *)
        nN = 20;
        (* list of the first nN coefficients divided by
            factorials *)
        an = ParallelTable[a[n]/n!, {n, nN}]

Out[1]:= {0, 0, 0, 1, 0, 2, 0, 9/2, 0, 12, 0, 112/3, 0, 130, 0,
     1961/4, 0, 5876/3, 0, 40871/5}
```

Listing 2. The coefficients of the partition function

```
In[2]:= (*the first nN coefficients NOT divided by factorials *)
        An = ParallelTable[A[n], {n, nN}];
        (* list of the coefficients of the partition function*)
        Yn = (Total /@ (Nest[MapThread[
           Join[#1, {#2}] &, {#, 1/(Length@#[[1]] + 1)
             Table[Sum[ Binomial[nn, l] An[[nn - l]]*
               #[[l, Length@#[[1]]]], {l,Length@#[[1]], nn -
                   1}], {nn, nN}]}] &, Partition[An, 1],
                   nN - 1])/Table[i!, {i, nN}])

Out[2]:= {0, 0, 0, 1, 0, 2, 0, 5, 0, 14, 0, 44, 0, 152, 0, 566,
     0, 2234, 0, 9228}
```

[1] L. Comtet, *Advanced Combinatorics: The Art of Finite and Infinite Expansions* (Reidel Publishing Company, Dordrecht, 1974).

[2] P. D. Beale, "Exact distribution of energies in the two-dimensional ising model," Phys. Rev. Lett. **76**, 78; DOI:10.1103/PhysRevLett.76.78 (1996).

[3] F. Wang and D. P. Landau, "Efficient, multiple-range random walk algorithm to calculate the density of states," Phys. Rev. Lett. **86**, 2050; DOI:10.1103/PhysRevLett.86.2050 (2001).

[4] D. Landau and K. Binder, *A Guide to Monte Carlo Simulations in Statistical Physics* (Cambridge University Press, New York, 2009).
